# Comparison of Opioids Prescribed by Advanced Practice Clinicians vs Surgeons After Surgical Procedures in the US

**DOI:** 10.1001/jamanetworkopen.2022.49378

**Published:** 2023-01-04

**Authors:** Caitlin R. Priest, Jennifer F. Waljee, Mark C. Bicket, Hsou-Mei Hu, Kao-Ping Chua

**Affiliations:** 1 Section of Plastic Surgery, Department of Surgery, University of Michigan Medical School, Ann Arbor; 2 Michigan Opioid Prescribing Engagement Network, University of Michigan Medical School, Ann Arbor; 3 Department of Anesthesiology, University of Michigan Medical School, Ann Arbor; 4 Susan B. Meister Child Health Evaluation and Research Center, Department of Pediatrics, University of Michigan Medical School, Ann Arbor; 5 Department of Health Management and Policy, University of Michigan School of Public Health, Ann Arbor

## Abstract

**Importance:**

Advanced practice clinicians (APCs), defined as nurse practitioners and physician assistants, are increasingly being incorporated into surgical teams. Despite this inclusion, there are no recent national data on the role of these clinicians in surgical opioid prescribing or the dosing of such prescriptions.

**Objective:**

To calculate the proportion of surgical opioid prescriptions written by APCs and to compare the total and daily dosages of these prescriptions with those written by surgeons.

**Design, Setting, and Participants:**

This cross-sectional study used the Optum’s De-Identified Clinformatics Data Mart, which contains deidentified claims from patients with private insurance and Medicare Advantage plans across the US. Adults and children who underwent 1 of 31 inpatient and outpatient surgical procedures from January 1, 2017, through November 30, 2019, were identified. The analysis was limited to procedures with 1 or more perioperative opioid prescriptions, defined as an opioid prescription dispensed within 3 days of the index date of surgery. Data were analyzed from April 1, 2021, to July 31, 2022.

**Exposures:**

Prescriber specialty.

**Main Outcomes and Measures:**

The outcome was the proportion of perioperative opioid prescriptions and refill prescriptions written by APCs. Linear regression was used to compare the total dosage of perioperative opioid prescriptions written by APCs vs surgeons measured in morphine milligram equivalents (MMEs). Models were adjusted for demographic characteristics, comorbidities, opioid-naive status, year of index date, hospitalization or observation status, surgical complications, and surgeon specialty. Analyses were conducted at the procedure level, and patients with multiple procedures were included.

**Results:**

Analyses included 628 197 procedures for 581 387 patients (358 541 females [57.1%]; mean [SD] age, 56 [18] years). Overall, APCs wrote 119 266 (19.0%) of the 628 197 perioperative opioid prescriptions and 59 679 (25.1%) of the 237 740 refill prescriptions. Perioperative opioid prescriptions written by APCs had higher total dosages compared with those written by surgeons (adjusted difference, 40.0 MMEs; 95% CI, 31.3-48.7 MMEs). This difference persisted in a subgroup analysis limited to opioid-naïve patients (adjusted difference, 15.7 MMEs; 95% CI, 13.9-17.5 MMEs).

**Conclusions and Relevance:**

In this cross-sectional analysis, one-fifth of perioperative opioid prescriptions and one-quarter of refill prescriptions were written by APCs. While surgeons wrote most perioperative opioid prescriptions that were intended for perioperative analgesia, higher total dosages from APCs suggest that opioid stewardship initiatives that support the role of APCs may be warranted.

## Key Points

**Question:**

What proportion of opioid prescriptions for surgical procedures is written by advanced practice clinicians (APCs)?

**Findings:**

In this cross-sectional study of national claims data for 628 197 surgical procedures involving 581 387 adults and children, one-fifth of perioperative opioid prescriptions and one-quarter of refill prescriptions were written by APCs. Perioperative opioid prescriptions written by APCs had higher total dosages compared with those written by surgeons.

**Meaning:**

Findings of this study suggest that opioid stewardship initiatives that support the role of APCs in surgical opioid prescribing may be warranted.

## Teaser

This cross-sectional study assesses postsurgical opioid prescribing practice and dosing by clinician type and surgical specialty.

## Introduction

Opioid prescriptions written for surgery frequently exceed the amount needed by patients,^1^ leading to leftover opioids that can be a source for misuse.^2^ Opioid exposure after surgery is associated with an increased risk of opioid-related adverse events, such as persistent opioid use.^3,4,5^ As the risk of these adverse events increases with opioid prescription size,^6,7^ reducing excessive opioid prescribing after surgery could mitigate opioid-related morbidity.

Advanced practice clinicians (APCs), defined as nurse practitioners and physician assistants, are increasingly being incorporated into surgical teams.^8,9,10,11,12,13^ Despite this inclusion, there are no recent national data on the role of these clinicians in surgical opioid prescribing or the dosing of their surgical opioid prescriptions. In one of the few studies on these topics, APCs wrote one-fifth of opioid prescriptions within 30 days of surgery among adult opioid-naïve patients and private insurance in Michigan who underwent surgical procedures between 2012 and 2015.^14^ Moreover, the mean total dosage of opioid prescriptions written by APCs was 18% higher than the dosage written by surgeons.^14^ While important, this study used older data from a single state; examined a small number of surgical procedures; and did not include children, older adults, or adults with opioid exposure, who represent approximately 40% of patients undergoing surgery.^14,15^

In this study, we used national commercial insurance and Medicare Advantage claims to identify adults and children who underwent 1 of 31 surgical procedures between 2017 and 2019, including those who did and did not have prior opioid exposure. The objective was to calculate the proportion of surgical opioid prescriptions written by APCs and to compare the total and daily dosages of these prescriptions with those written by surgeons.

## Methods

### Data Source

In April 2022, we conducted a cross-sectional analysis of 2017 to 2019 data from the Optum’s De-Identified Clinformatics Data Mart, which contains claims from commercially insured and Medicare Advantage plan enrollees across the US. The University of Michigan Medical School Institutional Review Board deemed this study exempt from review and waived the informed consent requirement because the data were deidentified. We followed the Strengthening the Reporting of Observational Studies in Epidemiology (STROBE) reporting guideline for reporting observational studies.

Data elements included patient demographic characteristics and provider taxonomy codes, which were based on specialty information reported by clinicians to plans. We used these codes to classify clinicians as APCs (nurse practitioners or physician assistants), surgeons (colorectal, general, hand, neurosurgery, obstetrics and gynecology, oral and maxillofacial, orthopedic, otolaryngology, pediatric, plastic, thoracic, transplant, urology, or vascular), or other prescribers. Surgeons included residents and fellows who self-reported a surgical specialty. To identify pharmacy claims for dispensed opioid prescriptions that were reimbursed by insurance, we used a list of national drug codes published by the Centers for Disease Control and Prevention.^16^ This list excluded opioid cough and cold medications and buprenorphine products indicated for opioid use disorder (eAppendix 1 in Supplement 1).

### Study Cohort

Using *Current Procedural Terminology* codes, we identified children and adults who underwent 1 of 31 common inpatient and outpatient surgical procedures from January 1, 2017, through November 30, 2019 (eAppendix 2 in Supplement 1). The index date was the date of surgery for outpatient procedures without subsequent hospitalization or the date of hospital discharge for inpatient procedures and outpatient procedures with subsequent hospitalization.

We excluded procedures if patients lacked continuous enrollment from 365 days before to 30 days after the index date, had additional major surgical procedures during the 30 days before to 30 days after the index date, had inpatient procedures for which the procedure date was not between the admission and discharge dates of the hospitalization (to account for potential data error), were hospitalized for more than 30 days after surgery, were not discharged home, did not reside in 1 of the 50 states or the District of Columbia, or had missing data on zip code. We further excluded procedures that were not associated with 1 or more dispensed opioid prescriptions on or within 3 days of the index date (perioperative opioid prescription) and procedures for which the clinician who was listed on the surgical claim was not a surgeon (to facilitate analyses by specialty). Analyses were conducted at the procedure level. Patients could undergo multiple procedures.

### Study Variables

We defined a refill as an opioid prescription dispensed after a perioperative opioid prescription and within 30 days of the index date. Procedures could be associated with 0, 1, or more than 1 refill prescription. We calculated the proportion of perioperative opioid prescriptions and refill prescriptions written by surgeons, APCs, and other prescribers. For each perioperative opioid prescription, we measured the total dosage in morphine milligram equivalents (MMEs), a standardized measure of opioid potency, by multiplying strength with quantity with published MME conversion factors.^16^ For the small number of procedures with multiple opioid prescriptions dispensed within 3 days of the index date, we summed MMEs across prescriptions, thus ensuring that each procedure was associated with only 1 perioperative opioid prescription. We calculated the daily dosage of perioperative opioid prescriptions by dividing the total dosage by days supplied, a variable that is calculated by pharmacies and directly reported in the Clinformatics database. Furthermore, we calculated the total and daily dosages of the first refill among procedures with 1 or more refill prescriptions. We analyzed only the first refill to facilitate comparisons between prescriber types.

Covariates included patient age; sex; payer type (commercial insurance vs Medicare Advantage); region of residence (based on the US Census); urban vs rural residence (based on zip code); year of index date; opioid-naive status (defined as the lack of opioid dispensing in the 365 days to 1 day before the index date); hospitalization or observation status; surgical complications (eg, respiratory failure, pulmonary embolism, and surgical-site infection); Charlson Comorbidity Index (categorized as 0, 1, 2, 3, or ≥4, with higher scores indicating greater number of comorbidities); smoking status; presence of mental health disorders, substance use disorders, musculoskeletal pain disorders, and other pain disorders in patients; and surgeon specialty, a potential indicator of the specialty of the individual supervising APCs on the surgical team. We did not include procedure type to avoid collinearity with surgeon specialty. The eAppendix 3 in Supplement 1 provides additional details on measuring covariates.

### Statistical Analysis

Using χ^2^ tests, we calculated the proportion of perioperative opioid prescriptions and refill prescriptions written by surgeons, APCs, and other prescribers. We repeated this analysis among subgroups that were defined by patient opioid-naive status, demographic characteristics, and year of index date and by surgeon specialty.

To identify factors associated with receiving a perioperative opioid prescription from an APC vs another prescriber type, we fitted a logistic regression model that adjusted for all covariates. To identify factors associated with receiving 1 or more refill prescriptions vs 0 refill prescriptions from an APC, we limited to procedures with 1 or more refill prescriptions and fitted a similar logistic regression model. In both of these models, we calculated average marginal effects to allow for interpretation of coefficients as absolute percentage point changes in probability.

To compare the total and daily dosages of perioperative opioid prescriptions between prescriber types, we fitted linear models that adjusted for all covariates. To compare the total and daily dosages of the first refill between prescriber types, we fitted similar models except that we also controlled for the number of days between the index date and the first refill.

In a subgroup analysis, we compared the total and daily dosages of perioperative opioid prescriptions and first refill prescriptions between surgeons and APCs only among opioid-naive patients, for whom the opioids prescribed within 30 days of surgery were highly likely to be for perioperative analgesia. In contrast, the opioids prescribed within 30 days of surgery for patients with opioid exposure could also be for continuation of long-term opioid therapy. In all models, we used SEs clustered at the patient level. Analyses were performed from April 1, 2021, to July 31, 2022, using SAS, version 9.4 (SAS Institute Inc), Stata, version 15.1/MP (StataCorp LLC), and 2-sided hypothesis tests with α = .05.

To ascertain whether the findings would generalize to a broader range of surgical procedures, we repeated the main analyses when including all major surgical procedures listed in the Surgery Flags Software for Services and Procedures (Agency for Healthcare Research and Quality).^17^ Additionally, to account for the possibility that APCs may be more likely to serve as the primary opioid prescribers for invasive procedures that require higher opioid dosage, we controlled for procedure type rather than surgeon specialty when comparing the total and daily dosages of perioperative opioid prescriptions and first refill prescriptions between surgeons and APCs. If 2 or more of the 31 included procedures occurred on the same index date, we assigned the patient to the procedure with the higher prevalence in the sample.

## Results

### Sample Characteristics

Among the 3 879 549 procedures that met the inclusion criteria, 628 197 were included in the final sample (eAppendix 4 in Supplement 1), which involved 581 387 patients. Mean (SD) patient age at the time of the procedure was 56 (18) years, and the analysis included 358 541 females (57.1%) and 269 656 males (42.9%); 372 039 patients (59.2%) were privately insured (Table 1). The most common surgeon specialties were orthopedic surgery (39.6%) and general surgery (29.2%). The 3 most frequent procedures were total knee arthroplasty, inguinal femoral hernia repair, and cholecystectomy (eAppendix 2 in Supplement 1).

**Table 1.** Proportion of Postoperative Opioid Prescriptions Written by APCs vs Other Prescriber Types

Characteristics	Total No. of prescriptions	Prescriptions by prescriber type, No. (%)				
		Surgeons	APCs	Other prescribers	Physician assistants	Nurse practitioners
No. of prescriptions	628 197	459 001 (73.1)	119 266 (19.0)	49 930 (7.9)	88 742 (14.1)	30 524 (4.9)
Patients						
Age, y						
0-17	16 679	14 600 (87.5)	1357 (8.1)	722 (4.3)	831 (5.0)	526 (3.2)
18-34	71 824	60 279 (83.9)	6544 (9.1)	5001 (7.0)	4465 (6.2)	2079 (2.9)
35-54	167 528	130 781 (78.1)	24 787 (14.8)	11 960 (7.1)	18 479 (11.0)	6308 (3.8)
55-64	130 881	91 527 (69.9)	28 652 (21.9)	10 702 (8.2)	21 657 (16.5)	6995 (5.3)
≥65	241 285	161 814 (67.1)	57 926 (24.0)	21 545 (8.9)	43 310 (17.9)	14 616 (6.1)
Sex						
Male	269 656	191 658 (71.1)	56 802 (21.1)	21 196 (7.9)	42 695 (15.8)	14 107 (5.2)
Female	358 541	267 343 (74.6)	62 464 (17.4)	28 734 (8.0)	46 047 (12.8)	16 417 (4.6)
Payer type						
Commercial insurance	372 039	286 689 (77.1)	60 374 (16.2)	24 976 (6.7)	45 227 (12.2)	15 147 (4.1)
Medicare Advantage	256 158	172 312 (67.3)	58 892 (23.0)	24 954 (9.7)	43 515 (17.0)	15 377 (6.0)
Region of residence						
Northeast	46 954	24 714 (52.6)	19 382 (41.3)	2858 (6.1)	15 543 (33.1)	3839 (8.2)
Midwest	178 563	127 147 (71.2)	41 494 (23.2)	9922 (5.6)	28 508 (16.0)	12 986 (7.3)
South	310 587	247 472 (79.7)	32 283 (10.4)	30 832 (9.9)	23 312 (7.5)	8971 (2.9)
West	92 093	59 668 (64.8)	26 107 (28.3)	6318 (6.9)	21 379 (23.2)	4728 (5.1)
Urban vs rural residence						
Urban	539 464	392 134 (72.7)	103 305 (19.1)	44 025 (8.2)	77 511 (14.4)	25 794 (4.8)
Rural	88 733	66 867 (75.4)	15 961 (18.0)	5905 (6.7)	11 231 (12.7)	4730 (5.3)
Opioid-naive status						
Yes	359 967	272 279 (75.6)	63 780 (17.7)	23 908 (6.6)	47 509 (13.2)	16 271 (4.5)
No	268 230	186 722 (69.6)	55 486 (20.7)	26 022 (9.7)	41 233 (15.4)	14 253 (5.3)
Year of index date						
2017	208 895	156 126 (74.7)	35 969 (17.2)	16 800 (8.0)	26 733 (12.8)	9236 (4.4)
2018	214 860	157 146 (73.1)	40 556 (18.9)	17 158 (8.0)	30 227 (14.1)	10 329 (4.8)
2019	204 442	145 729 (71.3)	42 741 (20.9)	15 972 (7.8)	31 782 (15.5)	10 959 (5.4)
Surgical complication						
Yes	47 160	26 317 (55.8)	11 996 (25.4)	8847 (18.8)	7465 (15.8)	4531 (9.6)
No	581 037	432 684 (74.5)	107 270 (18.5)	41 083 (7.1)	81 277 (14.0)	25 993 (4.5)
Hospitalization or observation status						
No hospitalization or observation	374 372	305 007 (81.5)	51 999 (13.9)	17 366 (4.6)	42 333 (11.3)	9666 (2.6)
Observation only	31 128	21 579 (69.3)	5807 (18.7)	3742 (12.0)	3911 (12.6)	1896 (6.1)
Hospitalization						
<7 d	198 737	121 328 (61.0)	54 622 (27.5)	22 787 (11.5)	38 704 (19.5)	15 918 (8.0)
7-30 d	23 960	11 087 (46.3)	6838 (28.5)	6035 (25.2)	3794 (15.9)	3044 (12.7)
CCI						
0	303 801	232 108 (76.4)	52 397 (17.2)	19 296 (6.4)	40 395 (13.3)	12 002 (4.0)
1	118 777	85 410 (71.9)	24 041 (20.2)	9326 (7.9)	18 033 (15.2)	6008 (5.1)
2	73 874	52 002 (70.4)	15 566 (21.1)	6306 (8.5)	11 399 (15.4)	4167 (5.6)
3	47 384	32 740 (69.1)	10 196 (21.5)	4448 (9.4)	7349 (15.5)	2847 (6.0)
≥4	84 361	56 741 (67.3)	17 066 (20.2)	10 554 (12.5)	11 566 (13.7)	5500 (6.5)
Tobacco use						
Yes	147 398	101 807 (69.1)	31 022 (21.0)	14 569 (9.9)	21 861 (14.8)	9161 (6.2)
No	480 799	357 194 (74.3)	88 244 (18.4)	35 361 (7.4)	66 881 (13.9)	21 363 (4.4)
Mental health disorder						
Yes	196 431	140 165 (71.4)	38 173 (19.4)	18 093 (9.2)	27 806 (14.2)	10 367 (5.3)
No	431 766	318 836 (73.8)	81 093 (18.8)	31 837 (7.4)	60 936 (14.1)	20 157 (4.7)
AUD or SUD						
Yes	77 543	54 092 (69.8)	15 073 (19.4)	8378 (10.8)	10 539 (13.6)	4534 (5.8)
No	550 654	404 909 (73.5)	104 193 (18.9)	41 552 (7.5)	78 203 (14.2)	25 990 (4.7)
Musculoskeletal pain						
Yes	469 000	327 593 (69.8)	103 951 (22.2)	37 456 (8.0)	79 389 (16.9)	24 562 (5.2)
No	159 197	131 408 (82.5)	15 315 (9.6)	12 474 (7.8)	9353 (5.9)	5962 (3.7)
Other pain disorder						
Yes	199 388	143 665 (72.1)	37 138 (18.6)	18 585 (9.3)	26 626 (13.4)	10 512 (5.3)
No	428 809	315 336 (73.5)	82 128 (19.2)	31 345 (7.3)	62 116 (14.5)	20 012 (4.7)
Surgeons						
Self-reported specialty						
Colorectal surgery	8058	6318 (78.4)	941 (11.7)	799 (9.9)	602 (7.5)	339 (4.2)
General surgery	183 620	143 312 (78.0)	21 432 (11.7)	18 876 (10.3)	14 274 (7.8)	7158 (3.9)
Hand surgery	2139	1852 (86.6)	173 (8.1)	114 (5.3)	151 (7.1)	22 (1.0)
Neurosurgery	32 897	17 947 (54.6)	11 468 (34.9)	3482 (10.6)	7337 (22.3)	4131 (12.6)
Obstetrics and gynecology	75 848	66 940 (88.3)	3699 (4.9)	5209 (6.9)	1598 (2.1)	2101 (2.8)
Oral and maxillofacial surgery	238	140 (58.8)	27 (11.3)	71 (29.8)	13 (5.5)	14 (5.9)
Orthopedic surgery	248 498	164 779 (66.3)	69 558 (28.0)	14 161 (5.7)	57 954 (23.3)	11 604 (4.7)
Otolaryngology	37 784	34 475 (91.2)	1490 (3.9)	1819 (4.8)	1034 (2.7)	456 (1.2)
Pediatric surgery	1079	724 (67.1)	225 (20.9)	130 (12.0)	58 (5.4)	167 (15.5)
Plastic surgery	7750	5769 (74.4)	923 (11.9)	1058 (13.7)	630 (8.1)	293 (3.8)
Thoracic surgery	11 844	4212 (35.6)	5665 (47.8)	1967 (16.6)	2942 (24.8)	2723 (23.0)
Transplant surgery	765	411 (53.7)	223 (29.2)	131 (17.1)	132 (17.3)	91 (11.9)
Urology	11 052	7862 (71.1)	2112 (19.1)	1078 (9.8)	1240 (11.2)	872 (7.9)
Vascular surgery	6625	4260 (64.3)	1330 (20.1)	1035 (15.6)	777 (11.7)	553 (8.3)

Abbreviations: APC, advanced practice clinician; AUD, alcohol use disorder; CCI, Charlson Comorbidity Index (categorized as 0, 1, 2, 3, or ≥4, with higher scores indicating greater number of comorbidities); SUD, substance use disorder.

### Role of APCs in Perioperative Opioid Prescriptions

Of the 628 197 perioperative opioid prescriptions, APCs wrote 119 266 (19.0%), surgeons wrote 459 001 (73.1%), and other prescribers wrote 49 930 (7.9%). Physician assistants wrote more perioperative opioid prescriptions (88 742 [14.1%]) than nurse practitioners (30 524 [4.9%]). Perioperative opioid prescriptions from other prescribers (n = 49 930) were most commonly written by internists (21 326 [42.7%]) and family medicine physicians (7375 [14.8%]).

As shown in Table 1, the proportion of perioperative opioid prescriptions written by APCs was higher when the procedures were for older patients and for patients residing in the Northeast (41.3%) compared with other regions (10.4%-28.3%). Among specialties, this proportion was lowest in otolaryngology (3.9%) and obstetrics and gynecology (4.9%) and highest in thoracic surgery (47.8%), neurosurgery (34.9%), and transplant surgery (29.2%). In adjusted models, several factors were associated with receiving a perioperative opioid prescription from APCs, including but not limited to older age, commercial insurance, residency outside of the South, urban residency, year of index date beyond 2017, admission to observation or the hospital, and surgeon specialty (Table 2). For example, patients were 29.1 (95% CI, 28.6-29.5) percentage points more likely to receive a perioperative opioid prescription from an APC if they resided in the Northeast compared with the South.

**Table 2.** Factors Associated With Receipt of Perioperative Opioid Prescriptions and Refill Prescriptions From APCs

Factors	Average marginal effects (95% CI)	
	Receipt of perioperative opioid prescription from APCs vs surgeons^a^	Receipt of ≥1 refills vs 0 refills from APCs^b^
**Patients**		
Age, y		
≥65	Reference	Reference
0-17	−4.2 (−5.0 to −3.4)	−5.6 (−8.3 to −2.9)
18-34	−3.6 (−4.1 to −3.2)	−4.4 (−5.6 to −3.2)
35-54	−1.9 (−2.3 to −1.6)	−0.9 (−1.7 to −0.2)
55-64	−1.1 (−1.5 to −0.8)	0.1 (−0.5 to 0.8)
Sex		
Male	Reference	Reference
Female	−0.2 (−0.4 to <0.01)	0.3 (−0.2 to 0.7)
Payer type		
Commercial insurance	Reference	Reference
Medicare Advantage	−2.2 (−2.5 to −1.9)	−3.2 (−3.8 to −2.6)
Region of residence		
South	Reference	Reference
Midwest	12.9 (12.7 to 13.2)	17.2 (16.6 to 17.7)
Northeast	29.1 (28.6 to 29.5)	25.8 (24.8 to 26.8)
West	18.3 (18.0 to 18.5)	25.4 (24.7 to 26.1)
Urban vs rural residence		
Rural	Reference	Reference
Urban	2.0 (1.7 to 2.2)	0.9 (0.3 to 1.5)
Opioid-naive status		
No	Reference	Reference
Yes	0.7 (0.5 to 0.9)	1.4 (0.9 to 1.8)
Year of index date		
2017	Reference	Reference
2018	2.0 (1.8 to 2.2)	2.7 (2.2 to 3.2)
2019	3.7 (3.5 to 3.9)	4.4 (3.9 to 5.0)
Surgical complication		
No	Reference	Reference
Yes	1.3 (1.0 to 1.7)	0.8 (0.0 to 1.5)
Hospital or observation status		
No hospital or observation	Reference	Reference
Observation only without hospitalization	9.4 (9.0 to 9.9)	2.7 (1.6 to 3.8)
Hospitalization		
<7 d	11.5 (11.3 to 11.8)	4.3 (3.8 to 4.8)
7-30 d	11.8 (11.2 to 12.4)	1.4 (0.3 to 2.5)
CCI		
0	Reference	Reference
1	−0.3 (−0.5 to −0.02)	−0.8 (−1.4 to −0.2)
2	0.3 (0.0 to 0.6)	−1.6 (−2.3 to −0.9)
3	−0.1 (−0.5 to 0.2)	−1.8 (−2.6 to −0.9)
≥4	−0.6 (−0.9 to −0.3)	−2.6 (−3.3 to −1.9)
Tobacco use		
No	Reference	Reference
Yes	0.6 (0.3 to 0.8)	1.1 (0.5 to 1.7)
Has mental health disorder		
No	Reference	Reference
Yes	0.2 (0.0 to 0.4)	0.6 (0.2 to 1.1)
Has AUD or SUD		
No	Reference	Reference
Yes	−0.7 (−1.0 to −0.4)	0.2 (−0.5 to 0.9)
Has musculoskeletal pain		
No	Reference	Reference
Yes	2.7 (2.4 to 3.0)	6.5 (5.5 to 7.5)
Has other pain disorder		
No	Reference	Reference
Yes	−0.3 (−0.5 to −0.1)	−0.3 (−0.7 to 0.2)
**Surgeons**		
Self-reported specialty		
General surgery	Reference	Reference
Colorectal surgery	−1.6 (−2.3 to −0.9)	−2.2 (−4.2 to −0.1)
Hand surgery	−0.5 (−2.1 to 1.1)	−3.5 (−7.4 to 0.5)
Neurosurgery	20.1 (19.5 to 20.6)	15.5 (14.6 to 16.5)
Obstetrics and gynecology	−8.4 (−8.6 to −8.2)	−6.9 (−7.7 to −6.0)
Oral and maxillofacial surgery	1.3 (−3.4 to 6.1)	−4.1 (−14.2 to 6.0)
Orthopedic surgery	13.1 (12.8 to 13.4)	12.5 (11.9 to 13.1)
Otolaryngology	−7.0 (−7.4 to −6.7)	−4.2 (−5.3 to −3.0)
Pediatric surgery	13.9 (11.1 to 16.7)	12.7 (−8.3 to 33.6)
Plastic surgery	2.1 (1.3 to 3.0)	2.2 (−0.2 to 4.7)
Thoracic surgery	24.4 (23.5 to 25.2)	12.5 (10.6 to 14.4)
Transplant surgery	13.0 (10.0 to 16.0)	7.6 (0.2 to 15.0)
Urology	2.9 (2.2 to 3.5)	−0.3 (−2.3 to 1.6)
Vascular surgery	5.7 (4.8 to 6.6)	4.5 (2.1 to 7.0)

Abbreviations: APC, advanced practice clinician; AUD, alcohol use disorder; CCI, Charlson Comorbidity Index (categorized as 0, 1, 2, 3, or ≥4, with higher scores indicating greater number of comorbidities); SUD, substance use disorder.

^a^ Represents the absolute percentage point difference in the probability of receiving a perioperative opioid prescription from an APC vs a surgeon if all patients had the level of the categorical variable in question vs the level of the reference group, holding all other covariates constant. Average marginal effects were derived from a multivariable logistic regression model.

^b^ Represents the absolute percentage point difference in the probability of receiving 1 or more refills vs 0 refills from an APC if all patients had the level of the categorical variable in question vs the level of the reference group, holding all other covariates constant. Average marginal effects were derived from a multivariable logistic regression model. Only procedures associated with 1 or more refills were included in this model.

### Role of APCs in Refill Prescriptions

Among the 628 197 procedures, 157 595 (25.1%) had 1 or more refill prescriptions. The 157 595 procedures had a total of 237 740 refill prescriptions, of which 124 853 (52.5%) were written by surgeons, 59 679 (25.1%) by APCs, and 53 208 (22.4%) by other prescribers. Similar to perioperative opioid prescriptions, the proportion of refill prescriptions written by APCs was higher among older patients and patients residing in the Northeast (41.6%) compared with the Midwest (32.2%), South (15.0%), and West (37.3%). Moreover, this proportion similarly varied widely by specialty, ranging from 10.0% in obstetrics and gynecology to 31.9% in neurosurgery (eAppendix 5 in Supplement 1).

Table 2 displays the adjusted association between covariates and the receipt of 1 or more refill prescriptions from APCs. In general, these associations were in the same direction as the corresponding associations between factors and the receipt of perioperative opioid prescriptions from APCs, although the magnitude of the associations often differed. For example, having a thoracic surgeon perform the procedure was associated with a 24.4 (95% CI, 23.5-25.2) percentage point higher probability of receiving a perioperative opioid prescription from an APC, but instead was associated with a 12.5 (95% CI, 11.9-13.1) percentage point higher probability of receiving 1 or more refill prescriptions from an APC.

### Dosing Characteristics of Perioperative Opioid Prescriptions and First Refill Prescriptions by Prescriber Type

Among perioperative opioid prescriptions, the mean total dosage was 260.2 MMEs from surgeons and 362.4 MMEs from APCs (adjusted difference [APCs minus surgeons], 40.0 MMEs; 95% CI, 31.3-48.7 MMEs) (Table 3). The magnitude of this difference was large in some specialties (eg, plastic surgery: 297 vs 187; adjusted difference, 111 MMEs [95% CI, 71-150 MMEs]) and nonexistent in others (eg, neurosurgery: 425 vs 424; adjusted difference, 1 MME [95% CI, –17 to 19 MMEs]) (Figure). The mean daily dosage was 44.5 MMEs from surgeons and 51.3 MMEs from APCs (adjusted difference, 2.5 MMEs; 95% CI, 2.2-2.8 MMEs).

**Table 3.** Adjusted Association Between Prescriber Type and Total and Daily Opioid Dosage of Perioperative Opioid Prescriptions and First Refill Prescriptions

Prescriber type	Perioperative opioid prescriptions (n = 628 197)				First refill prescriptions (n = 157 595)			
	Total dosage, MME		Daily dosage, MME		Total dosage, MME		Daily dosage, MME	
	Unadjusted mean	Adjusted difference (95% CI)^a^	Unadjusted mean	Adjusted difference (95% CI)^a^	Unadjusted mean	Adjusted difference (95% CI)^a^	Unadjusted mean	Adjusted difference (95% CI)^a^
**All procedures**								
Surgeons	260.2	Reference	44.5	Reference	353.7	Reference	46.1	Reference
APCs	362.4	40.0 (31.3 to 48.7)	51.3	2.5 (2.2 to 2.8)	525.5	126.2 (74.2 to 178.3)	52.4	3.6 (2.0 to 5.2)
Other prescribers	488.9	193.9 (151.5 to 236.3)	45.4	−0.4 (−1.8 to 0.9)	1191.0	742.5 (619.3 to 865.8)	50.2	5.9 (2.1 to 9.6)
**Procedures for opioid-naive patients**								
Surgeons	217.2	Reference	41.3	Reference	272.0	Reference	43.2	Reference
APCs	288.6	15.7 (13.9 to 17.5)	46.9	2.0 (1.8 to 2.2)	301.6	−4.0 (−8.0 to 0.1)	48.0	2.3 (1.8 to 2.8)
Other prescribers	200.3	−37.2 (−39.6 to −34.9)	38.4	−3.5 (−3.8 to −3.2)	241.2	−5.9 (−14.2 to 2.4)	34.1	−5.7 (−6.4 to −5.1)
**Procedures for patients with opioid exposure **								
Surgeons	323.1	Reference	49.2	Reference	406.6	Reference	47.9	Reference
APCs	447.3	73.6 (54.6 to 92.6)	56.3	3.2 (2.6 to 3.8)	644.3	201.4 (123.4 to 279.5)	54.8	4.4 (2.1 to 6.8)
Other prescribers	754.0	403.9 (321.9 to 486.0)	51.8	2.1 (−0.6 to 4.7)	1381.0	929.5 (773.6 to 1085.4)	53.4	8.8 (4.0 to 13.7)

Abbreviations: APC, advanced practice clinician; AUD, alcohol use disorder; MME, morphine milligram equivalent; SUD, substance use disorder.

^a^ Adjusted for age, sex, geographic region, payer type, rural or urban residency, smoking status, comorbidity, mental health disorder, AUD and SUD, pain disorder, year of index date, opioid-naive status, surgical complication, hospital or observation status, and specialty of surgeon.

**Figure.**
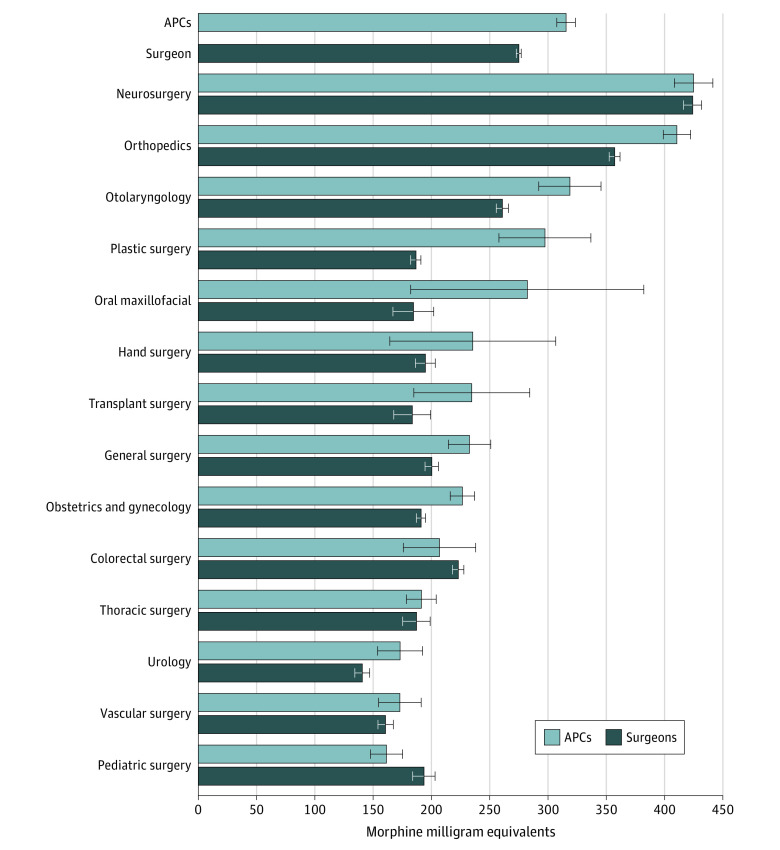
Adjusted Total Morphine Milligram Equivalents of Perioperative Opioid Prescriptions From Surgeons and Advanced Practice Clinicians (APCs), Overall and by Surgeon Specialty Specialty-specific values were derived from a linear regression model that included all covariates and an interaction term between prescriber type and surgeon specialty. Error bars represent 95% CIs.

Among first refill prescriptions, the mean total dosage was 353.7 MMEs from surgeons and 525.5 MMEs from APCs (adjusted difference, 126.2 MMEs; 95% CI, 74.2-178.3 MMEs). The mean daily dosage was 46.1 MMEs from surgeons and 52.4 MMEs from APCs (adjusted difference: 3.6 MMEs; 95% CI, 2.0-5.2 MMEs) (Table 3).

When limiting to opioid-naive patients, the difference in the total dosage of perioperative opioid prescriptions between APCs and surgeons was attenuated but persisted (adjusted difference, 15.7 MMEs; 95% CI, 13.9-17.5 MMEs). The difference in the daily dosage of perioperative opioid prescriptions was similarly attenuated (adjusted difference, 2.0 MMEs; 95% CI, 1.8-2.2 MMEs). In contrast to the overall analysis, there was no significant difference in the total dosage of first refill prescriptions between APCs and surgeons for opioid-naive status patients (adjusted difference, −4.0 MMEs; 95% CI, −8.0 to 0.1 MMEs), although the daily dosage remained higher (adjusted difference, 2.3 MMEs; 95% CI, 1.8-2.8 MMEs) (Table 3).

### Sensitivity Analysis

In the sensitivity analysis that included a broader range of surgical procedures, there were 841 083 procedures for 759 143 patients. Conclusions were similar to those of the main analysis. The proportion written by APCs was 17.8% of perioperative opioid prescriptions and 23.3% of first refill prescriptions compared with 19.0% and 25.1%, respectively, in the main analysis. The adjusted difference (APCs minus surgeons) was 58.8 (95% CI, 51.3-66.3) MMEs in the total dosage and 2.4 (95% CI, 2.2-2.7) MMEs in the daily dosage of perioperative opioid prescriptions, compared with 40.0 MMEs and 2.5 MMEs, in the main analysis. The adjusted difference was 197.4 (95% CI, 122.8-743.9) MMEs in the total dosage and 4.2 (95% CI, 1.8-6.5) MMEs in the daily dosage of first refill prescriptions compared with 126.2 MMEs and 3.6 MMEs, respectively, in the main analysis.

When controlling for procedure type rather than surgeon specialty, the adjusted difference was 22.9 (95% CI, 14.1-31.6) MMEs in the total dosage and 1.4 (95% CI, 1.1-1.7) MMEs in the daily dosage of perioperative opioid prescriptions, compared with 40.0 MMEs and 2.5 MMEs, respectively, in the main analysis. The adjusted difference in the total and daily dosages of the first refill prescriptions was virtually identical to the main analysis.

## Discussion

In this cross-sectional analysis of national claims data from children and adults who underwent 1 of 31 common surgical procedures between 2017 and 2019, APCs wrote one-fifth of perioperative opioid prescriptions and one-quarter of refill prescriptions. The mean total dosage of perioperative opioid prescriptions was higher when written by APCs rather than surgeons, even when accounting for patient and procedural factors, suggesting that the difference was not solely associated with case mix. While surgeons remain the primary prescriber of opioid prescriptions intended for perioperative analgesia, the findings highlight the crucial role of APCs in surgical opioid prescribing. Moreover, higher total dosages of perioperative opioid prescriptions from APCs suggest that surgical opioid stewardship initiatives focused on these clinicians may be warranted.

A previous analysis of 2012 to 2015 data from privately insured, opioid-naive adult patients in Michigan found that APCs wrote 19% of opioid prescriptions dispensed within 30 days of 18 surgical procedures.^14^ The present study adds to that analysis by differentiating between perioperative and refill prescriptions using recent national data; examining a wider variety of surgical procedures; and including adults, children, and Medicare Advantage patients with opioid exposure. Additionally, the present study provided new data on variation in the role of APCs in surgical opioid prescribing. For example, we found that the proportion of perioperative opioid prescriptions and refill prescriptions written by APCs varied by surgeon specialty and was highest in the Northeast, perhaps reflecting the higher concentration of APCs in this region compared with other regions.^18^

The adjusted difference in the total dosage of perioperative opioid prescriptions written by APCs vs surgeons was 40 MMEs, corresponding to 8 pills containing 5 mg hydrocodone. Because the sample included patients with opioid exposure, one potential explanation for this difference is that some perioperative opioid prescriptions written by APCs were intended for continued management of chronic pain rather than perioperative pain. However, when limiting the analysis to opioid-naïve patients, we found the adjusted difference was still 15.7 MMEs, corresponding to approximately 3 pills containing 5 mg hydrocodone. These findings suggest that APCs do provide higher dosages of opioid prescriptions to manage acute pain immediately after surgery.

The adjusted difference in the total dosage of first refill prescriptions written by APCs vs surgeons was 126.2 MMEs, corresponding to approximately 25 pills containing 5 mg hydrocodone. However, there was no difference when limiting the analysis to opioid-naive patients. The findings suggest that some first refill prescriptions written by APCs in the overall analysis may have been for chronic pain and do not necessarily support the notion that APCs provide higher total dosages of refill prescriptions compared with surgeons to manage postsurgical pain.

Daily dosages were higher when perioperative opioid prescriptions and first refill prescriptions were written by APCs rather than by surgeons. These differences persisted when limiting the analysis to opioid-naive patients. However, the small magnitude of the differences, approximately one-half of a pill containing 5 mg hydrocodone, suggests they may not be clinically significant.

Collectively, results from these analyses suggest that any APC-specific surgical opioid stewardship initiatives may need to focus on ensuring that the total dosage of perioperative opioid prescriptions matches patient need. Such initiatives may be more important in certain surgical specialties than in others. For example, the proportion of surgical opioid prescriptions written by APCs varied widely by specialty surgery, as did the difference in the total dosage of perioperative opioid prescriptions written by APCs and surgeons.

In this study, 7.9% of perioperative opioid prescriptions and 22.4% of refill prescriptions were written by clinicians other than APCs or surgeons. Some of these prescriptions were likely written for reasons other than surgery. For example, these prescriptions typically had high total dosages and were predominantly written by internists and family medicine physicians, suggesting that many of these prescriptions were written for chronic pain. A caveat, however, is that internists and family medicine physicians serving as hospitalists could also prescribe opioids to patients who were admitted after surgery. Surgical opioid stewardship initiatives ideally would have the resources to include the full range of clinicians who might prescribe opioids for perioperative analgesia.

### Limitations

This study has limitations. First, findings may not generalize to patients without insurance or patients covered by fee-for-service Medicare or Medicaid. Second, misclassification of surgeon specialty is possible, although specialty was self-reported by clinicians. Third, similar to other claims-based studies, we relied on the timing of opioid prescription dispensing relative to surgery to identify surgical opioid prescriptions, as the Clinformatics database does not directly report prescription indication. Thus, some perioperative opioid prescriptions and refill prescriptions in this study may not have been intended for surgical pain. Results from the subgroup analysis limited to opioid-naive patients are likely less prone to such misclassification.

## Conclusions

In this cross-sectional study of claims from children and adults who underwent surgical procedures, one-fifth of perioperative opioid prescriptions and one-quarter of refill prescriptions after surgery were written by APCs. Future studies are needed to identify which quality improvement initiatives can best support the role of APCs in surgical opioid prescribing.

## Notes

Supplement 1. **eAppendix 1.** List of Opioid Analgesics Included **eAppendix 2.** Frequency of Procedures in the Sample **eAppendix 3.** Details on Methodology for Measuring Covariates **eAppendix 4.** Inclusion and Exclusion Criteria **eAppendix 5.** Proportion of Refills Accounted for By Advanced Practice Clinicians (APCs) Versus Other Clinicians

Supplement 2.
Data Sharing Statement
