# Supplementary material for: Comparison of Opioids Prescribed by Advanced Practice Clinicians vs Surgeons After Surgical Procedures in the US
Source: JAMA Netw Open. 2023 Jan 4;6(1):e2249378. doi: 10.1001/jamanetworkopen.2022.49378 (PMC9857656; doi:10.1001/jamanetworkopen.2022.49378)
Supplement: Supplement 1. — eAppendix 1. List of Opioid Analgesics Included eAppendix 2. Frequency of Procedures in the Sample eAppendix 3. Details on Methodology for Measuring Covariates eAppendix 4. Inclusion and Exclusion Criteria eAppendix 5. Proportion of Refills Accounted for By Advanced Practice Clinicians (APCs) Versus Other Clinicians [file jamanetwopen-e2249378-s001.pdf]

Supplemental Online Content

Priest CR, Waljee JF, Bicket MC, Hu HM, Chua KP. Comparison of opioids prescribed by advanced practice clinicians vs surgeons after surgical procedures in the US. *JAMA Netw Open*. 2023;6(1):e2249378. doi:10.1001/jamanetworkopen.2022.49378

eAppendix 1. List of Opioid Analgesics Included

eAppendix 2. Frequency of Procedures in the Sample

eAppendix 3. Details on Methodology for Measuring Covariates

eAppendix 4. Inclusion and Exclusion Criteria

eAppendix 5. Proportion of Refills Accounted for By Advanced Practice Clinicians (APCs) Versus Other Clinicians

This supplemental material has been provided by the authors to give readers additional information about their work.

© 2023 Priest CR et al. *JAMA Network Open.*

eAppendix 1. List of opioid analgesics included

Buprenorphine
Butorphanol
Codeine
Dihydrocodeine
Fentanyl buccal or SL tablets, or lozenge/troches
Fentanyl film or oral spray
Fentanyl nasal spray
Fentanyl patch
Hydrocodone
Hydromorphone
Levomethadyl
Levorphanol tartrate
Meperidine
Methadone
Morphine
Opium
Oxycodone
Oxymorphone
Pentazocine
Propoxyphene
Tapentadol
Tramadol

© 2023 Priest CR et al. *JAMA Network Open.*

eAppendix 2. Frequency of procedures in the sample

**Procedure**	**No. (%)**
Total knee arthroplasty	63,382 (10.1)
Inguinal femoral hernia repair	61,587 (9.8)
Cholecystectomy	49,697 (7.9)
Arthroscopic knee meniscectomy	44,652 (7.1)
Arthroscopy of shoulder	39,864 (6.3)
Cesarean section	38,638 (6.2)
Hysterectomy	34,897 (5.6)
Total hip arthroplasty	32,816 (5.2)
Arthrodesis of spine	31,725 (5.1)
Neuroplasty transposition	27,155 (4.3)
Laminectomy	24,851 (4.0)
Lumpectomy/mastectomy	18,419 (2.9)
Excision of skin lesions	17,748 (2.8)
Tonsillectomy and/or adenoidectomy	16,823 (2.7)
Appendectomy	15,956 (2.5)
Inferior turbinate resection or excision	15,641 (2.5)
Tendon sheath incision	13,771 (2.2)
Colectomy/proctectomy	11,611 (1.8)
Arthroplasty of shoulder	9,980 (1.6)
Coronary artery bypass graft	8,376 (1.3)
Debridement of wound	8,073 (1.3)
Hemorrhoid procedures	7,212 (1.1)
Thyroidectomy	5,666 (0.9)
Laparoscopic and open prostatectomy	5,256 (0.8)
Gastric bypass	4,866 (0.8)
Endarterectomy	4,382 (0.7)
Heart valve procedures	4,316 (0.7)
Nephrectomy	4,204 (0.7)
Small bowel resection/enterolysis	3,698 (0.6)
Skin grafts	2,032 (0.3)
Abdominal aortic aneurysm repair	903 (0.1)
TOTAL	628,197

© 2023 Priest CR et al. *JAMA Network Open.*

eAppendix 3. Details on methodology for measuring covariates

Tobacco Use was measured using ICD-10-CM diagnosis codes F17*, Z87891, Z720

Mental health and substance use disorders were measured using AHRQ's Clinical Classification System (CCS)

**Disorder**	**CCS Multi-level 2 Category**
Mental Health Disorders, including	
Anxiety Disorder	5.2
Mood Disorder	5.8
Adjustment Disorder	5.1
Suicide and Intentional Self-inflicted Injury	5.13
Disruptive Disorder	5.3; 5.7
Personality Disorder	5.9
Schizophrenia and Other Psychotic Disorders	5.10
Other Disorders	5.15
Drug & Substance Use Disorders	5.11; 5.12

Source: Clinical Classifications Software (CCS) for ICD-10-PCS (beta version). Healthcare Cost and Utilization Project (HCUP). November 2019. Agency for Healthcare Research and Quality, Rockville, MD. www.hcup-us.ahrq.gov/toolssoftware/ccs10/ccs10.jsp.

Pain disorders were measures using these ICD-10-CM diagnosis codes:

**Pain Disorder**	**Diagnosis Code**
Musculoskeletal pain (back Pain, neck pain, arthritis/joint pain)	**Back Pain**<br>M40.0*,M40.2*,M41.00,M41.04-M41.08, M41.20,M41.24-M41.27,M41.3*, M41.80, M41.84-M41.87,M41.9,M43.00,M43.04-M43.10,M43.14-M43.19,M43.27,M43.28, M46.40,M46.44-M46.48, M47.10,M47.14-M47.16,M47.814-M47.819, M48.00, M48.04-M48.08, M48.10, M48.14-M48.20, M48.24-M48.27,M48.30,M48.34-M48.38,M48.9,M51.04-M51.06, M51.44-M51.47, M51.84-51.87, M51.9,M53.2*4-M53.2*9,M53.3,M53.9,M54.04-M54.08,M54.14-M54.18, M54.3*-M54.6, M54.89,M54.9,M96.1-M96.3, M96.5, M99.02-M99.04,M99.83,M99.84, Q76.2,Q76.4*, S22.009A, S23.101A, S23.111A, S23.121A, S23.123A, S23.131A, S23.133A, S23.141A, S23.143A, S23.151A, S23.153A, S23.161A, S23.163A, S23.171A, S23.3XXA, S23.8XXA, S23.9XXA, S32.009A, S32.10XA, S32.2XXA, S33.101A, S33.2XXA, S33.5XXA, S33.6XXA, S33.8XXA, S33.9XXA<br><br>**Neck pain**<br>M41.02,M41.03,M41.22,M41.23,M41.82, M41.83,M43.01,M43.02,M43.03, M43.11,M43.12, M43.13,M43.6,M47.11,M47.12, M47.13, M47.811,M47.812,M47.813,M48.01,M48.02, M48.03, M50.0*,M50.2*,M50.8*,M50.9*,M53.0,M53.1, M53.82,M54.01, M54.02,M54.03, M54.11, M54.12, M54.13, M54.2, M99.01, S13.101A, S13.111A, S13.121A, S13.131A, S13.141A, S13.151A,

© 2023 Priest CR et al. *JAMA Network Open.*

	S13.161A, S13.171A, S13.181A, S13.4XXA, S13.8XXA<br><br>**Arthritis and joint pain**<br>M00.*,M01.*,M02.*,M05.*,M06.*,M08.*,M11.*,M12.*,M13.*,M14.*,M15.*, M16.*, M17.*,M18.*, M19.*,M20.*,M21.*,M22.*, M23.*, M24.*,M25.*,M32.*,M33.*,M34.*,M36.*,M42.*,M61.*, M62.*, M65.*,M66.*,M67.*,M70.*, M71.*,M72.*,M75.*, M76.*, M77.*,M80.*,M81.*,M84.*,M85.*, M86.*, M87.*,M88.*,M89.*,M90.*,M91.*,M92.*,M93.*,M94.*,M95.*,M40.1*, M40.4*, M40.5*, M41.4*, M41.5*,M43.8*,M60.0*,M60.1*,M60.2*,M79.6*,M43.9*,M35.1*,M35.2*, M35.3*,M35.4*,M35.5*, M35.6*, M35.7*, M35.8*,M35.9*,M96.4*,R25.2*,R26.2*,R29.4*,R29.898, S12.000K,S12.001K, S12.100K,S12.101K,S12.200K, S12.201K, S12.300K,S12.301K,S12.400K,S12.401K,S12.500K,S12.501K, S12.600K,S12.601K,S42.009K,S42.009P,S42.209K, S42.209P, S42.91SK,S42.92SK,S82.009P,S82.009Q, S82.009R,S92.819K,S92.819P,S92.909K,S92.909P,S92.919K,S92.919P,S99.209K, S99.209P, S99.219K, S99.219P,S99.229K,S99.229P,S99.239K,S99.239P,S99.249K,S99.249P,S99.299K,S99.299P,M48.40*A, M48.41*A,M48.42*A,M48.43*A,M48.44*A,M48.45*A,M48.46*A,M48.47*A,M48.48*A,M48.50*A, M48.51*A,M48.52*A, M48.53*A,M48.54*A,M48.55*A,M48.56*A,M48.57*A,M48.58*A, S02.91*K, S02.92*K, 42.90*K,S52.90*K,S62.90*K, S72.90*K, S82.90*K,S52.90*M,S72.90*M,S82.90*M, S52.90*N, S72.90*N,S82.90*N,S52.90*Q,S72.90*Q,S82.90*Q, S52.90*R,S72.90*R, S82.90*R, S42.90*P,S42.91*P,S42.92*P,S52.90*P,S62.90*P,S72.90*P,S82.90*P,S22.9**K,S32.9**K
Other Pain Disorders	G43.*, R51.*,K58.*,K59.*,N80.*,K30.*,M60.9*,M79.1*,M79.9*,'M60.8*,K22.4*,M26.6*,H93.1*, M35.0*, M54.81*, N30.10*,N30.11*,R07.82*,R07.89*,N94.81*,R53.82*,G47.00*,H04.129,G44201, G44209

Surgical complications were measured using these ICD-10-CM diagnosis codes.

**Surgical Complication**	**Diagnosis Code**
Pulmonary Failure	J96*
Pneumonia	J13*, J148*, J15*, J16*, J17*, J188
Myocardial Infarction	I21*
DVT, PE	I26*, I80*, I82*
Renal Failure	N17*
Hemorrhage	R58*, L760*, I976*, D780*, K91840, I9742*
GI Bleeding	K922*
Surgical Site Infection	T81*, T798*, K651*, L7634*

© 2023 Priest CR et al. *JAMA Network Open.*

eAppendix 4. Inclusion and exclusion criteria

Included 31 surgical procedures from January 1, 2017 through November 30, 2019 (3,879,549 procedures)
Excluded procedures lacking continuous enrollment from 365 days prior to index date through 30 days after index date (1,017,243 procedures)
Excluded procedures for patients who had another surgery within 30 days (797,690 procedures)
Excluded inpatient procedures for which the procedure date did not fall between the admission and discharge date (8 procedures)
Excluded procedures for patients who were hospitalized for more than 30 days (8,627 procedures)
Excluded procedures for patients who were not discharged to home (97,089 procedures)
Excluded procedures for patients who were did not reside in the 50 states or D.C. and those who did not have a zip code on file (e.g. '00000' or blank) (41,753 procedures)
Excluded procedures for patients who didn't fill opioid prescription 0 – 3 days after discharge (1,127,931 procedures)
Excluded procedures for which the rendering provider was not a surgeon (161,011 procedures)
Final cohort: 628,197 procedures

© 2023 Priest CR et al. *JAMA Network Open.*

eAppendix 5. Proportion of refills accounted for by advanced practice clinicians (APCs) versus other clinicians

	All prescribers, No.	Surgeon, No. (%)	APCs, No. (%)	Other prescribers, No. (%)	Physician assistant No. (%)	Nurse practitioner No. (%)
**OVERALL**	237,740	124,853 (52.5%)	59,679 (25.1%)	53,208 (22.4%)	43,406 (18.3%)	16,273 (6.8%)
**Age**						
0-17	1,565	1,204 (76.9%)	209 (13.4%)	152 (9.7%)	130 (8.3%)	79 (5.0%)
18-34	12,895	8,690 (67.4%)	2,015 (15.6%)	2,190 (17.0%)	1,362 (10.6%)	653 (5.1%)
35-54	56,297	30,398 (54.0%)	12,697 (22.6%)	13,202 (23.5%)	8,882 (15.8%)	3,815 (6.8%)
55-64	65,314	32,326 (49.5%)	17,704 (27.1%)	15,284 (23.4%)	12,853 (19.7%)	4,851 (7.4%)
65+	101,669	52,235 (51.4%)	27,054 (26.6%)	22,380 (22.0%)	20,179 (19.8%)	6,875 (6.8%)
**Sex**						
Male	98,587	51,061 (51.8%)	25,440 (25.8%)	22,086 (22.4%)	18,715 (19.0%)	6,725 (6.8%)
Female	139,153	73,792 (53.0%)	34,239 (24.6%)	31,122 (22.4%)	24,691 (17.7%)	9,548 (6.9%)
**Payer Type**						
Commercial	113,488	64,250 (56.6%)	28,639 (25.2%)	20,599 (18.2%)	21,051 (18.5%)	7,588 (6.7%)
Medicare Advantage	124,252	60,603 (48.8%)	31,040 (25.0%)	32,609 (26.2%)	22,355 (18.0%)	8,685 (7.0%)
**Geographic Region**						
Northeast	14,739	5,899 (40.0%)	6,132 (41.6%)	2,708 (18.4%)	4,619 (31.3%)	1,513 (10.3%)
Midwest	67,868	33,966 (50.0%)	21,836 (32.2%)	12,066 (17.8%)	15,243 (22.5%)	6,593 (9.7%)
South	117,378	68,931 (58.7%)	17,622 (15.0%)	30,825 (26.3%)	12,359 (10.5%)	5,263 (4.5%)
West	37,755	16,057 (42.5%)	14,089 (37.3%)	7,609 (20.2%)	11,185 (29.6%)	2,904 (7.7%)
**Urban/rural residence**						
Urban	202,684	107,064 (52.8%)	51,027 (25.2%)	44,593 (22.0%)	37,615 (18.6%)	13,412 (6.6%)
Rural	35,056	17,789 (50.7%)	8,652 (24.7%)	8,615 (24.6%)	5,791 (16.5%)	2,861 (8.2%)
**Opioid-naïve**						
Yes	72,912	45,337 (62.2%)	19,794 (27.1%)	7,781 (10.7%)	15,268 (20.9%)	4,526 (6.2%)
No	164,828	79,516 (48.2%)	39,885 (24.2%)	45,427 (27.6%)	28,138 (17.1%)	11,747 (7.1%)
**Year of index date**						
2017	81,804	44,121 (53.9%)	18,552 (22.7%)	19,131 (23.4%)	13,712 (16.8%)	4,840 (5.9%)
2018	79,014	41,218 (52.2%)	20,267 (25.6%)	17,529 (22.2%)	14,795 (18.7%)	5,472 (6.9%)
2019	76,922	39,514 (51.4%)	20,860 (27.1%)	16,548 (21.5%)	14,899 (19.4%)	5,961 (7.7%)
**Surgical complication**						
Yes	26,288	11,601 (44.1%)	5,882 (22.4%)	8,805 (33.5%)	3,797 (14.4%)	2,085 (7.9%)
No	211,452	113,252 (53.6%)	53,797 (25.4%)	44,403 (21.0%)	39,609 (18.7%)	14,188 (6.7%)
**Hospitalization/observation status**						
No hospitalization/observation	103,132	54,039 (52.4%)	23,163 (22.5%)	25,930 (25.1%)	16,749 (16.2%)	6,414 (6.2%)
Observation only	11,209	6,213 (55.4%)	2,507 (22.4%)	2,489 (22.2%)	1,835 (16.4%)	672 (6.0%)
Hospitalization <7 days	110,959	59,139 (53.3%)	31,535 (28.4%)	20,285 (18.3%)	23,407 (21.1%)	8,128 (7.3%)
Hospitalization 7-30 days	12,440	5,462 (43.9%)	2,474 (19.9%)	4,504 (36.2%)	1,415 (11.4%)	1,059 (8.5%)
**Charlson Comorbidity Index**						

© 2023 Priest CR et al. *JAMA Network Open.*

0	92,528	53,008 (57.3%)	24,710 (26.7%)	14,810 (16.0%)	18,759 (20.3%)	5,951 (6.4%)
1	51,809	27,286 (52.7%)	13,566 (26.2%)	10,957 (21.1%)	9,879 (19.1%)	3,687 (7.1%)
2	31,479	16,076 (51.1%)	7,682 (24.4%)	7,721 (24.5%)	5,586 (17.7%)	2,096 (6.7%)
3	21,697	10,573 (48.7%)	5,227 (24.1%)	5,897 (27.2%)	3,658 (16.9%)	1,569 (7.2%)
4+	40,227	17,910 (44.5%)	8,494 (21.1%)	13,823 (34.4%)	5,524 (13.7%)	2,970 (7.4%)
**Tobacco use**						
Yes	78,567	37,511 (47.7%)	19,276 (24.5%)	21,780 (27.7%)	13,148 (16.7%)	6,128 (7.8%)
No	159,173	87,342 (54.9%)	40,403 (25.4%)	31,428 (19.7%)	30,258 (19.0%)	10,145 (6.4%)
**Mental health disorder**						
Yes	103,136	49,806 (48.3%)	25,134 (24.4%)	28,196 (27.3%)	17,577 (17.0%)	7,557 (7.3%)
No	134,604	75,047 (55.8%)	34,545 (25.7%)	25,012 (18.6%)	25,829 (19.2%)	8,716 (6.5%)
**Alcohol/substance use disorder**						
Yes	46,984	21,917 (46.6%)	10,885 (23.2%)	14,182 (30.2%)	7,299 (15.5%)	3,586 (7.6%)
No	190,756	102,936 (54.0%)	48,794 (25.6%)	39,026 (20.5%)	36,107 (18.9%)	12,687 (6.7%)
**Musculoskeletal pain**						
Yes	218,121	111,929 (51.3%)	57,215 (26.2%)	48,977 (22.5%)	42,057 (19.3%)	15,158 (6.9%)
No	19,619	12,924 (65.9%)	2,464 (12.6%)	4,231 (21.6%)	1,349 (6.9%)	1,115 (5.7%)
**Other pain disorder**						
Yes	93,433	45,311 (48.5%)	21,883 (23.4%)	26,239 (28.1%)	15,027 (16.1%)	6,856 (7.3%)
No	144,307	79,542 (55.1%)	37,796 (26.2%)	26,969 (18.7%)	28,379 (19.7%)	9,417 (6.5%)
**Specialty of surgeon performing procedure**						
Colorectal surgery	2,125	1,198 (56.4%)	268 (12.6%)	659 (31.0%)	146 (6.9%)	122 (5.7%)
General surgery	38,001	17,264 (45.4%)	5,752 (15.1%)	14,985 (39.4%)	2,989 (7.9%)	2,763 (7.3%)
Hand surgery	498	253 (50.8%)	56 (11.2%)	189 (38.0%)	37 (7.4%)	19 (3.8%)
Neurosurgery	20,019	8,118 (40.6%)	6,390 (31.9%)	5,511 (27.5%)	3,693 (18.4%)	2,697 (13.5%)
Obstetrics/gynecology	11,319	7,494 (66.2%)	1,132 (10.0%)	2,693 (23.8%)	512 (4.5%)	620 (5.5%)
Oral and maxillofacial surgery	59	24 (40.7%)	6 (10.2%)	29 (49.2%)	3 (5.1%)	3 (5.1%)
Orthopedic surgery	148,499	81,863 (55.1%)	43,176 (29.1%)	23,460 (15.8%)	34,508 (23.2%)	8,668 (5.8%)
Otolaryngology	7,745	5,285 (68.2%)	868 (11.2%)	1,592 (20.6%)	512 (6.6%)	356 (4.6%)
Pediatric surgery	29	7 (24.1%)	7 (24.1%)	15 (51.7%)	2 (6.9%)	5 (17.2%)
Plastic surgery	1,706	679 (39.8%)	297 (17.4%)	730 (42.8%)	133 (7.8%)	164 (9.6%)
Thoracic surgery	3,579	1,011 (28.2%)	964 (26.9%)	1,604 (44.8%)	466 (13.0%)	498 (13.9%)
Transplant surgery	213	65 (30.5%)	63 (29.6%)	85 (39.9%)	40 (18.8%)	23 (10.8%)
Urology	2,225	1,009 (45.3%)	348 (15.6%)	868 (39.0%)	186 (8.4%)	162 (7.3%)
Vascular surgery	1,723	583 (33.8%)	352 (20.4%)	788 (45.7%)	179 (10.4%)	173 (10.0%)

APC – advanced practice clinician

© 2023 Priest CR et al. *JAMA Network Open.*
