# Supplementary material for: Comparison of Opioids Prescribed by Advanced Practice Clinicians vs Surgeons After Surgical Procedures in the US
Source: JAMA Netw Open. 2023 Jan 4;6(1):e2249378. doi: 10.1001/jamanetworkopen.2022.49378 (PMC9857656; doi:10.1001/jamanetworkopen.2022.49378)
Supplement: Supplement 2. — Data Sharing Statement [file jamanetwopen-e2249378-s002.pdf]

Data Sharing Statement

Priest. Comparison of Opioids Prescribed by Advanced Practice Clinicians vs Surgeons After Surgical Procedures in the US. *JAMA Netw Open*. Published January 04, 2023. doi:10.1001/jamanetworkopen.2022.49378

Data
Data available: No

Additional Information
Explanation for why data not available: Data are proprietary are cannot be shared
